# An In Vivo Study in Rat Femurs of Bioactive Silicate Coatings on Titanium Dental Implants

**DOI:** 10.3390/jcm9051290

**Published:** 2020-04-29

**Authors:** Giulia Brunello, Lisa Biasetto, Hamada Elsayed, Elia Sbettega, Chiara Gardin, Anna Scanu, Simone Carmignato, Barbara Zavan, Stefano Sivolella

**Affiliations:** 1 Department of Management and Engineering, University of Padova, Stradella San Nicola 3, 36100 Vicenza, Italy; lisa.biasetto@unipd.it (L.B.); elia.sbettega@phd.unipd.it (E.S.); simone.carmignato@unipd.it (S.C.); 2 Department of Neurosciences, Dentistry Section, University of Padova, Via Giustiniani 2, 35128 Padova, Italy; stefano.sivolella@unipd.it; 3 Department of Industrial Engineering, University of Padova, Via F. Marzolo 9, 35131 Padova, Italy; hamada.elsayed@unipd.it; 4 Ceramics Department, National Research Centre, El-Bohous Street, Cairo 12622, Egypt; 5 Department of Medical Sciences, University of Ferrara, Via Fossato di Mortara 70, 44121 Ferrara, Italy; chiara.gardin@unife.it (C.G.); barbara.zavan@unife.it (B.Z.); 6 Maria Cecilia Hospital, GVM Care & Research, 48033 Cotignola, Italy; 7 Rheumatology Unit, Department of Medicine-DIMED, University of Padova, Via Giustiniani 2, 35128 Padova, Italy; anna.scanu@unipd.it

**Keywords:** implant, osseointegration, bioactive ceramic coating, sphene, micro-CT

## Abstract

Silica-based ceramics have been proposed for coating purposes to enhance dental and orthopedic titanium (Ti) implant bioactivity. The aim of this study was to investigate the influence of sphene-based bioceramic (CaO.TiO_2_.SiO_2_) coatings on implant osseointegration in vivo. Sphene coatings were obtained from preceramic polymers and nano-sized active precursors and deposited by an automatic airbrush. Twenty customized Ti implants, ten sphene-coated and ten uncoated rough implants were implanted into the proximal femurs of ten Sprague-Dawley rats. Overall, cortical and cancellous bone-to-implant contact (BIC) were determined using micro-computed tomography (micro-CT) at 14 and 28 days. Moreover, peri-implant bone healing was histologically and histomorphometrically evaluated. The white blood cell count in the synovial fluid of the knee joints, if present, was also assessed. No difference in the BIC values was observed between the sphene-coated and uncoated implants, overall and in the two bone compartments (*p* > 0.05). Delamination of the coating occurred in three cases. Consistently with micro-CT data, the histological evaluation revealed no differences between the two groups. In addition, no synovial fluid could be collected on the test side, thus confirming sphene biocompatibility. In conclusion, sphene coating was found to be a suitable material for biomedical applications. Further studies are needed to improve coating adhesion to the implants.

## 1. Introduction

Osseointegration, which is defined as the close contact between the implant and the surrounding living bone, is a fundamental condition for long-term implant success [1]. In order to foster and accelerate new bone formation at the bone–implant interface, several surface modifications of titanium (Ti) implants have been proposed [2,3].

Micro- and nano-roughening of Ti machined implants have been demonstrated to enhance the implant osseointegration. To that aim, both additive techniques, consisting in coating deposition procedures, as well as subtractive treatments (e.g., grit-blasting, sandblasting acid-etching, electropolishing, mechanical polishing, and laser micro-texturing) have been introduced [4,5]. The main advantage of additive approaches for surface modification is the possibility of incorporating bioactive molecules, species and drugs, and thereby creating the surface topography which could be attractive for osteoblast differentiation, adhesion and osteogenic activity. Bioactive coatings appear to be a promising strategy to enhance implant osseointegration [6,7]. Even though one of the main drawbacks of coatings consists of their chemical stability, bioactive coatings have been shown to mimic the biochemical milieu and architecture of bone tissue to a greater extent than uncoated Ti surfaces [5].

Hydroxyapatite, Ca_5_(PO_4_)_3_(OH), coatings applied on metallic implants have been extensively investigated, showing a positive effect on osseointegration compared to uncoated implants [8,9,10]. However, the poor adhesion of hydroxyapatite (HA) coatings to metallic substrates, leading to the failure of the implants by cracking and delamination of the coatings, has hampered their clinical application [11,12].

A new class of bioactive ceramics, known as silica-based ceramics, has recently gained considerable importance in biomedical applications as coating materials [6,13,14,15,16,17,18].

Silica-based ceramics, such as CaSiO_3_ (wollastonite) and Ca–M–Si (M = Mg, Zn, Ti, Zr), possess mechanical strength, especially fracture toughness, and bioactivity generally higher than that of HA ceramics. Contrary to calcium phosphate ceramics, such as HA, they are characterized by the presence of Si in their chemical composition, which has been demonstrated to contribute to their improved bioactivity [19]. In addition, the incorporation of elements (such as Zn, Mg, Ti, Zr and Sr) into calcium silicate ceramics has shown great potential to further promote their orthopedic applications. The addition of these elements improved the mechanical characteristics and controlled the dissolution rates, which provided an indication for stability and bioactivity (i.e., apatite formation and mineralization) [20].

However, it is still a relatively unexplored area of research, with limited in vivo data associated for adequate preliminary in vitro characterization [6].

Silicate ceramic coatings deposited onto Ti-based substrates have been demonstrated to promote cell attachment and proliferation [21,22,23,24,25]. Furthermore, when compared to HA-coatings, various silica-based coatings (i.e., akermanite (Ca_2_MgSi_2_O_7_), bredigite (Ca_7_Mg(SiO_4_)_4_), hardystonite (Ca_2_ZnSi_2_O_7_), sphene (CaTiSiO_5_), Sr-substituted hardystonite) have shown superior in vitro behavior [16,18,22,25]. Higher bone-to-implant contact values have also been reported for Ti-6Al-4V implants with hardystonite and Sr-substituted hardystonite coatings than for HA-coated implants after 12 weeks of healing in dog femur [25]. In conclusion, we could state that silica-based ceramics, compared to HA-modified implants, have a wide range of chemical compositions that allow to control the degradation rate and increase the bioactivity and mechanical properties, which could be considered as some limitations of HA-modified implants.

Among several silica-based ceramic coatings, of particular interest are the sphene-based (CaO.TiO_2_.SiO_2_) coatings, due to their improved adhesion to the substrates compared to HA coatings [26,27]. Sphene ceramic coatings, produced by the preceramic polymer processing route and applied on Ti plates by airbrushing, have exhibited high chemical stability and good adhesion to the substrates [15,21,28].

In vitro investigations confirmed that sphene ceramic coatings were able to support cell adhesion and proliferation [21,22]. Moreover, a significantly higher cell proliferation and alkaline phosphate activity were observed on sphene coatings than on HA-coated and uncoated substrates [22].

Animal studies on rats’ femurs are considered valid models for assessing implant osseointegration [29], usually measuring the parameter “bone-to-implant contact” (BIC) on histological images/sections [30]. The bone–implant interface can also be investigated with micro-computed tomography (micro-CT), which enables the 3D evaluation of the bone surrounding the implants and does not preclude further assessments afterwards [31,32,33,34,35]. This method overcomes the limits of histological analysis, such as the fact that a single histological section may not be representative of the bone apposition over the whole implant [36], and the fact that it is hard to obtain serial sectioning of bony specimens containing little metal implants positioned in small animals such as mice [37,38,39] and rats [40,41].

Synovial fluid (SF) aspiration is considered a useful tool for the diagnosis of joint diseases [42,43]. An excessive pathologic amount of SF in proximity to an implant can reveal the presence of inflammation as the host response to the implantable biomaterials [44,45,46,47,48]. In addition, SF analysis could be helpful in the assessment of the degree of local inflammation.

It was hypothesized that the surface modification via sphene coating would enhance the implant osseointegration. In order to prove this hypothesis, an in vivo study in the proximal femur of rats was carried out. Uncoated rough Ti implants were used as controls.

The primary aim of the present experimental study was to compare the BIC values assessed by micro-CT at two different time points of two rough titanium implant surfaces, uncoated and coated with sphene ceramic.

The secondary aims were the comparison between the test and control groups of the following: (a) a histological analysis of peri-implant bone healing; (b) the degree of the local inflammation through total white blood cell counting in the knee joint SF at the time of the sacrifice.

## 2. Experimental Section

### 2.1. Samples Preparation and Characterization

**Figure 1:**
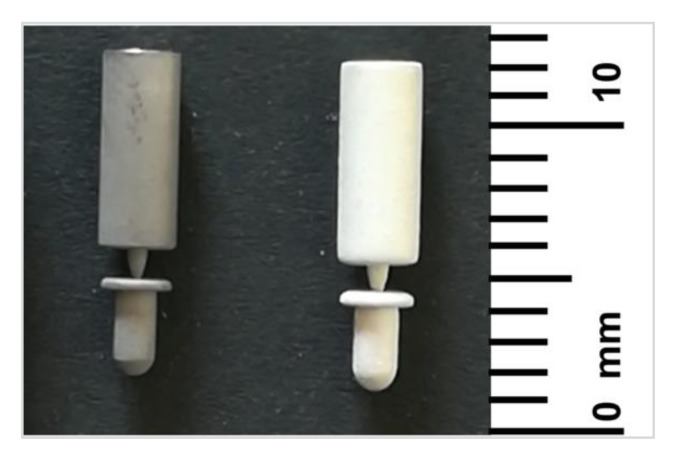
Customized uncoated (**left**) and sphene-coated (**right**) implants.

Customized cylindrical Ti implants, 1.45 mm in diameter and 3 mm in length, were used in the present research. The implants were characterized by a ZirTi^®^ surface, sand-blasted with zirconium oxide and etched with mineral acids [49]. The implant design was featured by a coronal extension (carrier), connected to the implant itself, for the easy handling of the implant during the coating deposition and implant insertion as shown in Figure 1.

Sphene coating on Ti implants was developed by the preceramic polymer processing route as extensively described in previous studies [15,21], using preceramic polymer SILRES^®^MK, isopropanol and nano-sized CaCO_3_ (90 nm) and TiO_2_ (25 nm) powders as raw materials. The composition of the suspension is reported in Table 1.

Briefly, the suspension was homogenized by sonication (15 min) and subsequently positioned into an automatic airbrush (Prona-RA-C2, PronaTools, Toronto, Canada). During the coating deposition, the solution was maintained homogenous by magnetic stirring.

**Figure 2:**
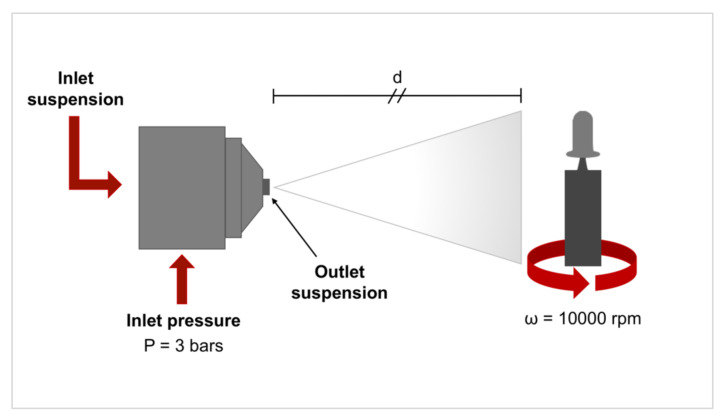
Schematic representation of the coating system composed of an automated airbrush and a rotatable implant holder. The customized implant, on the right, is characterized by the implant itself (in light grey) and by a coronal portion (in dark grey) for carrying the implant and which is easily detachable after the implant insertion. The main coating parameters are reported: inlet pressure P, rotating speed ω and the nozzle to implant distance.

Before the coating process, the Ti implants were ultrasonically cleaned in acetone, alcohol and deionized water for 10 min for each step, and then air-dried. The implants were secured to a rotating substrate holder, composed of a clamping system and a DC 3V micromotor (CX.KR280-2865, Commex, Pianiga, Italy). The implants were rotated at high speed (10000 rpm) and the coating was applied on the implant surface. The optimized parameters in terms of distance, pressure and deposition time were adopted after the preliminary optimization tests. The pressure was set at 3 bars and the airbrush nozzle of 1 mm in diameter was maintained at a specific distance from the implant (35 cm). A coating time of 1 s was selected. A schematic overview of the coating systems is reported in Figure 2.

After spraying the coating, the coated implants were left to dry and afterwards heat-treated in static air at 950 °C for 1 hour, with a heating rate of 5 °C/min.

Both the uncoated and sphene-coated ZirTi implants were tested in vitro for cytotoxicity evaluation and in vivo to investigate the influence of the surface modification on the implant osseointegration. Henceforth, the two implanted groups will be identified as the uncoated and the sphene-coated implants. Before the in vitro and in vivo experiments, all the implants were individually sterilized in a double package by dry autoclavation for 20 min at 121 °C.

The surface topography of both the uncoated and sphene-coated implants was investigated using an optical 3D profilometer (Sensofar S Neox, Barcelona, Spain) in confocal mode. A 20× objective (numerical aperture 0.45, field of view 877 µm × 660 µm) was used, with a spatial sampling of 0.65 µm and an optical resolution of 0.31 µm. Each implant was individually positioned with the cylinder axis in parallel to the sample table. An area covering half of the implant was scanned. Squared areas of 1 mm × 1 mm, extracted from the scanned surface, were examined. The areal surface texture parameters Sa and Sz were measured from the extracted areas in accordance with ISO 25178-2 [50], using the evaluation software MountainsMap^®^ (Digital Surf, Besançon, France).

The coating thickness and porosity were analyzed using a metrological X-ray computed tomography (CT) system Nikon MCT 225 (Nikon Metrology, Tring, UK). The parameters used for the CT scanning of the coated and uncoated implants before the surgery are listed in Table 2.

Three-dimensional image reconstruction was performed with CT Pro 3D software (Nikon Metrology, Tring, UK). For the elaboration of CT volumetric voxel data, the data analysis and visualization, the software VGStudio MAX 3.2 (Volume Graphics GmbH, Heidelberg, Germany) was used.

In the reconstructed volumetric voxel data, the segmentation between the region of interest (ROI) corresponding to the sphene coating (ROI_C_) and the ROI corresponding to the Ti implant (ROI_I_) was executed based on the different grey values of the voxels representing the different materials. For the calculation, the portion of the implant corresponding to the part which would be inserted into the bone was selected, cropping ROI_C_ 150 μm apically from the head of the implant. Within this volume, the mean coating thickness and porosity were calculated.

### 2.2. Toxicity

#### 2.2.1. Cells

The mesenchymal stem cells (MSCs) line C3H10T1/2 cells were purchased from ATCC (Manassas, VA, USA) and seeded at a density of 4 × 10^5^ cells/piece in cDMEM, which consisted of Dulbecco’s modified eagle medium (DMEM) (Lonza S.r.l., Milano, Italy), supplemented with 10 vol % Fetal Bovine Serum (FBS) (Bidachem-Spa, Milano, Italy) and 1 vol % Penicillin/Streptomycin (P/S) (EuroClone, Milano, Italy). The 3D cultures were incubated at 37 °C and 5% CO_2_ for 7 days, with media changes every 2 days. The control conditions were represented by cells cultured on tissue culture plates (TCP) in the cDMEM for the same culturing time.

#### 2.2.2. Lactate Dehydrogenase (LDH) Activity

The lactate dehydrogenase (LDH) activity was measured using a specific LDH Activity Assay Kit (Sigma-Aldrich) at 7 days of cell culture onto the samples. All the conditions were tested in duplicate. The culture medium was reserved to determine extracellular LDH activity. The intracellular LDH activity was estimated after the cells lysis with the assay buffer contained in the kit. All the samples were incubated with a supplied reaction mixture, resulting in a product whose absorbance was measured at 450 nm using the Victor 3 (Perkin Elmer, Milano, Italy) plate reader.

#### 2.2.3. Hemolysis Assay

The blood compatibility of the implants was evaluated by the hemolysis assay performed following the standard practices set forth in ASTM F756. Briefly, rabbit blood was diluted with phosphate-buffered saline (PBS) and used to cover a single test sample. For the negative control, a portion of high-density polyethylene (HDPE) was used. For the positive control, sterile water for injection (SWFI) was used. The extraction conditions were 50 °C for 72 h for all samples. Following the incubation, the tubes were centrifuged for 15 min at 800 g. A 1 ml aliquot of the resulting supernatant from the test materials and the negative and positive controls were added to 1 ml of Drabkin’s reagent (Sigma-Aldrich) and incubated at room temperature for 15 min. The reaction product between hemoglobin and Drabkin’s reagent was a cyano derivative that was quantified by measuring the absorbance at 540 nm with a multilabel plate reader (Victor 3). The hemolysis index (HI) was then calculated using the mean absorbance value (OD) for each group as follows: HI (%) = (OD (test material) − OD (negative control))/(OD (positive control) − OD (negative control)) X 100%. The samples were considered as non-hemolytic if the HI was 2% or less.

### 2.3. Surgical Procedure

This study was carried out in strict accordance with the recommendations in the EU Directive 2010/63/EU for animal experiments. All the experimental procedures were approved by the Animal Ethic Committee of Padova University and by the Italian Ministry of Health (No. 441/2018-PR). The study complies with the ARRIVE (Animals in Research: Reporting In Vivo Experiments) guidelines for reporting animal research [51].

Ten healthy 25-week-old male Sprague-Dawley rats, weighing approximately 300–350 g, were used in this study. All the animals were acclimated for one week prior to the surgery under climate-controlled conditions and fed with a standard laboratory diet and water ad libitum at the Experimental Surgery Center of the University of Padova. All the surgeries were performed by a single operator (G.B.).

The surgical procedure was performed under general anesthesia using sevoflurane 3% and 1.5%–2%, for induction and maintenance, respectively. Tramadol (5 mg/kg) was given for perioperative pain control.

Following anesthesia, the animals were positioned in supine position, the surgical fields were shaved and the skin was disinfected with 10 % povidone–iodine.

Each rat underwent a bilateral implantation of the sphene-coated implant on one femur and of the uncoated implant (control) on the contralateral one. The implant side was randomly assigned. The randomized treatment codes were enclosed in non-transparent sealed envelopes, opened sequentially after the animals were anesthetized.

**Figure 3:**
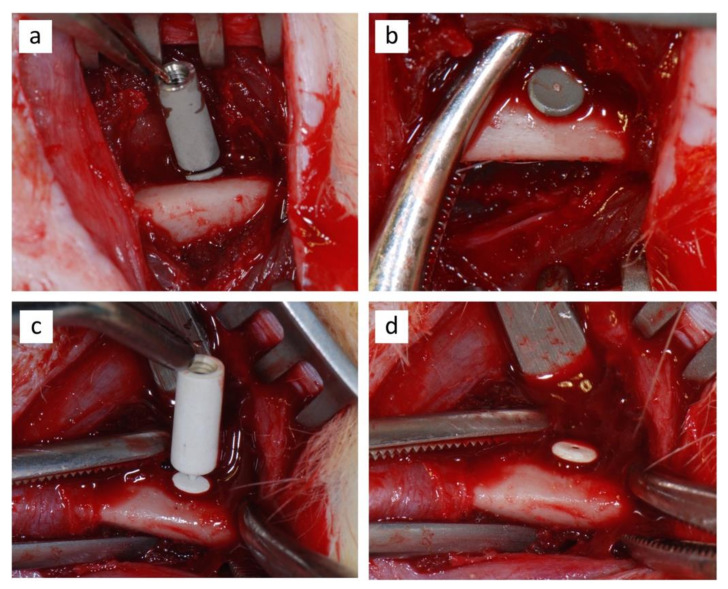
Surgical procedure: the positioning of the uncoated (**a**) and the sphene-coated implants (**c**) in the rats’ femurs, followed by the removal of respective carriers after the implant placement (**b**,**d**).

On each side, the proximal aspect of the femur was exposed via a lateral skin incision, followed by blunt dissection of the muscles. The implant sites were prepared under constant irrigation with sterile saline using a tungsten carbide round bur for the low-speed handpiece. The holes were slightly underprepared in order to achieve primary stability. The implants were placed into the proximal epiphysis of the bilateral femurs of each rat by press-fitting (Figure 3a,c). At this point the carriers were gently detached from the implants and removed (Figure 3b,d).

A primary-intention closure was obtained with the double-layer suture technique. The muscles were carefully sutured with 4-0 Vicryl (Ethicon, Inc., Somerville, NJ, USA) and the skin was closed with 3-0 Vicryl. Postoperative analgesia was achieved with the administration of tramadol (5 mg/kg) twice daily for 5 days. Cefazolin (1 mg/kg) was administered intra-muscularly for 5 days to avoid wound infections.

Five animals were sacrificed respectively after 14 and 28 days of healing, by an overdose of anesthesia. After the sacrifice, the synovial fluid was immediately collected from the rat knee joints. Finally, the femurs were dissected out and fixed in 4% phosphate-buffered formalin, pH 7, for the micro-CT examinations and subsequently, for histological analysis.

### 2.4. Micro-CT Analysis

For the micro-CT analysis, the metrological X-ray CT system Nikon MCT 225 (Nikon Metrology, Tring, UK) was used, as previously described in Section 2.1. Since the samples were conserved in 10% formalin solution, they were carefully retrieved from the container and fixed using a low-density rigid foam support, which was then positioned onto the rotary table. The samples were positioned with respect to the X-ray beam in order to minimize the presence of image artefacts. Before each scan, the samples were left inside the temperature-controlled CT cabinet for at least 1 h at 20 °C for thermal and mechanical stabilization.

The CT-scanning parameters used for the acquisition of the images of the implants after the sacrifice are summarized in Table 3.

**Figure 4:**
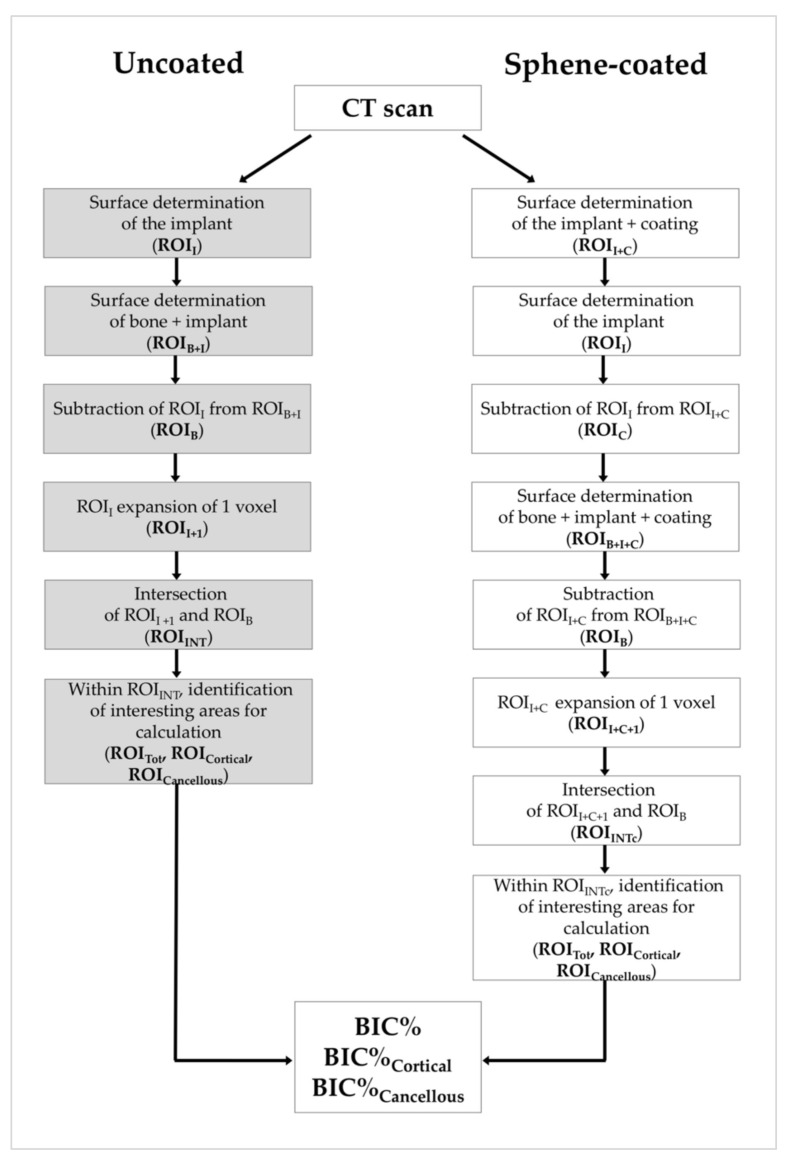
Workflow of the micro-computed tomography (micro-CT) analysis. BIC%: Bone to Implant Contact. BIC%_Cortical_ and BIC%_Cancellous_: BIC% obtained in cortical and cancellous bone zones, respectively. ROI: Region of Interest. ROI_I_—ROI implant; ROI_B_—ROI bone; ROI_B+I_—ROI bone + implant; ROI_I+1_—ROI implant + 1 voxel; ROI_INT_—ROI intersection; ROI_TOT_—ROI total; ROI_Cortical_—ROI cortical; ROI_Cancellous_—ROI cancellous; ROI_I+C_—ROI implant + coating; ROI_C_—ROI coating; ROI_I+C_—ROI implant + coating; ROI_B+I+C_—ROI bone + implant + coating; ROI_I+C+1_—ROI implant + coating + 1 voxel; ROI_INTc_—ROI intersection coating.

The reconstruction procedure was performed as indicated in Section 2.1. The volumetric data were elaborated following the procedure described in Figure 4.

**Figure 5:**
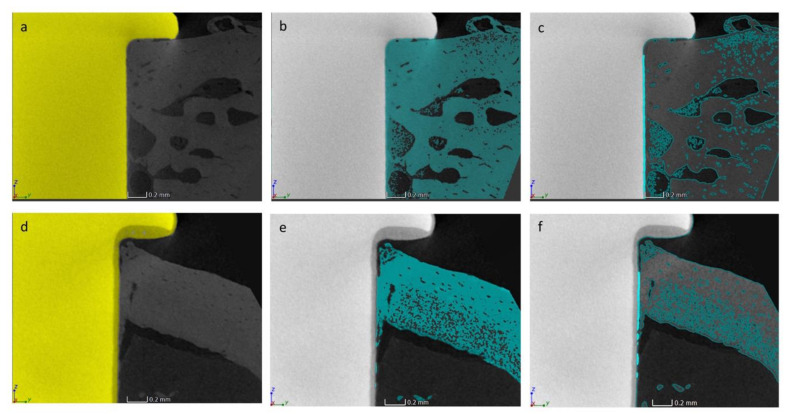
Representative micro-CT transverse cross-sectional views of the uncoated (**a**–**c**) and the sphene-coated (**d**–**f**) Ti implants inserted in the rat femur after 28 days of healing with the indicated selection of the ROI_I_ and ROI_I+C_ in yellow (**a**,**d**, respectively) and ROI_B_ in light blue (**b**,**e**). ROI_INT_ (**c**) and ROI_INTc_ (**f**) are shown indicated in fluorescent blue.

Based on the different grey values of the voxels corresponding to the different materials under investigation, different ROIs were identified, corresponding to the Ti implant (ROI_I_), the bone (ROI_B_) and for the sphene-coated implant, the coating (ROI_C_). As illustrated in Figure 5, the intersection between the bone and the implant (ROI_INT_) was computed for the uncoated samples (Figure 5c), and the intersection between the bone and the coating (ROI_INTc_) was computed for the sphene-coated samples (Figure 5f). The 3D bone to implant contact value (BIC%) was thus calculated from the analysis of ROI_INT_ and ROI_INTc_, which represent the contact zones between the bone and the implant (starting from 150 μm under the head of the implant, in order to avoid image artifacts induced by the presence of the implant head).

In order to investigate the effects of the coating on the implant osseointegration in the two different bone compartments (i.e., cortical and cancellous bone), within the ROI_INT_ and ROI_INTc_, sub-ROIs perpendicular to the long axis of the implant, 0.3 mm in height, were randomly drawn, one for each bone compartment. The BIC% obtained in these zones will hereafter be referred to as BIC%_Cortical_ and BIC%_Cancellous_. A clear description of the method is reported in the supplementary material file (Figures S1 and S2).

### 2.5. Histological Processing

After micro-CT analysis, the implants were carefully retrieved from the bone and the femurs were fixed in 4% phosphate-buffered formalin, pH 7, for 10 days, then transferred to a solution of 70% ethanol until processing. The samples were dehydrated with increasing concentrations of alcohol, up to 100%. The specimens were then sectioned along the longitudinal axis of the implant using a diamond disc at about 150 mm thick cross-section and prepared by grinding to approximately 50–60 µm thickness. The sections were finally stained with hematoxylin and eosin (H&E) and Masson’s trichrome staining for the histomorphometric analysis by means of optical microscopy.

### 2.6. Histological Assessment

The histomorphometric analysis was carried out on 3 sections per sample chosen randomly and observed under a Leitz Orthoplan light microscope (LM, Leica Microsystem Inc., Bannockburn, IL, USA) equipped with a computerized image analyzer system (Qwin, Leica Microsystem Imaging Solution Ltd, Cambridge, UK). From each section, 5 images were taken. The standard region of interest (ROI) was determined in a region of 1218 μm × 898 μm (1.09 × 10^6^ μm^2^). Within the ROI, the bone growth and maturation were examined using the Leica Qwin software. The area was analyzed for the presence or absence of polymorphonuclear cells, phagocytic cells, fibroblasts, endothelial cells, collagen fibers and new bone in order to evaluate bone healing in the control and tested groups at both time points. Each parameter was scored from 0 (absence) to 3 (abundantly present).

### 2.7. Synovial Fluid (SF) Analysis

The SF was aspirated from both the knee joints of each rat. If present, the volume of the aspirate was recorded, and the sample was analyzed for total white blood cell (WBC) count (WBC/mm^3^) to assess the degree of inflammation of the joints. For WBC/mm^3^ < 2.000, the SF was considered a non-inflammatory fluid without any stratification within that range. On the other hand, for the values above this threshold, it was designated as an inflammatory fluid and the increased levels of WBC/mm^3^ were associated with a higher degree of inflammation [43,52].

### 2.8. Statistical Analysis

Sample size calculation was based on reasonable assumptions from similar studies [53,54,55,56,57,58,59]. A sample size of 5 animals (in a paired design) was required to have an 80% chance of detecting, as significant at the 5% level, a standardized effect size of 2 in the primary outcome measure. The total sample size was 10 animals (5 animals were randomly sacrificed after 14 days of healing and 5 animals were randomly sacrificed after 28 days of healing). The continuous data were expressed as the mean and standard deviation (SD) and the categorical data as frequency and percentage. The continuous data were compared between the two study arms using a paired Student’s *t* test, while categorical data using the McNemar test. The BIC data were compared between 14 and 28 days using the Student’s *t*-test. A *p*-value less than 0.05 was considered statistically significant. The data analysis was performed using R 3.5 (R Foundation for Statistical Computing, Vienna, Austria).

## 3. Results

### 3.1. Surface and Coating Analysis

Surface roughness was investigated by means of confocal microscopy (see Section 2.1). Uncoated implants presented Sa and Sz values of 2.55 μm and 50.74 μm respectively, whereas higher values were found for the sphene-coated surfaces (Sa = 3.38 μm and Sz = 59.06 μm).

**Figure 6:**
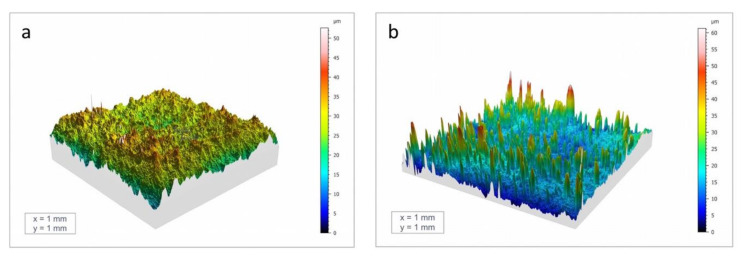
Three-dimensional surface topography of the uncoated implant (**a**) and the sphene-coated implant (**b**).

An increase in surface roughness was found after the coating deposition. As shown in Figure 6, the surface topography of the sphene coating was characterized by the presence of numerous peaks distributed on the whole surface, which were absent on the uncoated ZirTi surface. The yellow and red peaks in Figure 6B correspond to the edges of the white agglomerates detected by SEM observations and investigations using a stylus profilometer reported in a previous work [21], which were mainly composed of bioceramic sphene phase (CaO.TiO_2_.SiO_2_).

The porosity analysis was performed by micro-CT, finding no porosity inside the sphene coating; however, the presence of porosity or defects smaller than the micro-CT voxel size cannot be excluded.

The mean coating thickness, measured by micro-CT on one sample before insertion, was 61 ± 13.4 μm. It can be assumed that the high variability of the coating thickness was due to an eccentricity effect during the coating deposition.

### 3.2. Toxicity

**Figure 7:**
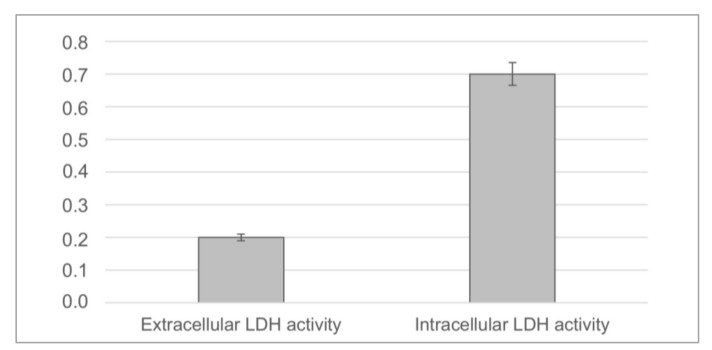
Cytotoxicity assay of mesenchymal stem cells (MSCs) cultured on CaO.TiO_2_.SiO_2_. Lactate dehydrogenase (LDH) leakage was measured at 7 days and reported as the optical density (OD) 490 nm/104 viable cells. The results are the mean of 2 samples from 3 different experiments ± SD. * *p* < 0.05.

The toxicity of the sphene-coated samples was evaluated by means of an LDH activity assay. In Figure 7, measures of its intracellular and extracellular levels are reported. As shown, we found a high level in the intracellular compartment and no detectable value in the extracellular environment. This low level of LDH activity detected in the culture medium confirmed that no cell damage had occurred due to a toxicity of the samples. Additionally, a hemolysis assay was carried to evaluate the blood compatibility of the sphene samples. The results are reported in Table 4, where it is clear that the hemolytic index (HI) was less than 2%, indicating the absence of any hemolytic activity of the tested material.

### 3.3. Clinical Observations in In Vivo Experiments

The surgical procedures were performed without any complications. The healing period was uneventful and all the animals survived during the experimental period. During the femur retrieval, at 14 and 28 days after the implant placement, no macroscopic signs of inflammation or adverse reactions were detected in the surrounding tissues. No implant dislocation had occurred and all the 20 implants were still in place at the moment of the sacrifice.

### 3.4. Micro-CT Analysis

The summary of the BIC%, BIC%_Cortical_ and BIC%_Cancellous_ values in the uncoated and the sphene-coated arms is reported in Table 5.

It should be noted that BIC% did not correspond to the sum of BIC%_Cortical_ and BIC%_Cancellous_, as described in the experimental part. Regarding BIC%, the values were slightly higher in the sphene-coated samples at both time points (uncoated vs. sphene-coated: *p* = 0.84 and *p* = 0.80 after 14 and 28 days, respectively).

The highest BIC% value was observed in sphene-coated samples after 28 days of healing (60.4 ± 17.7). Compared to the 14-day time point, there was a non-significant trend toward an increase in BIC% over time within each group (*p* = 0.41 for uncoated; *p* = 0.40 for sphene-coated).

The influence of the implant surface modification on the bone healing in the cortical portion of the bone was also investigated. As for BIC%, no statistically significant differences were found between the two groups at both time points (*p* = 0.35 and *p* = 0.39 after 14 and 28 days, respectively). The highest mean BIC%_Cortical_ was registered in the uncoated group after 28 days from the implant placement.

Even though an increase in BIC%_Cortical_ could be observed over time in uncoated samples, no statistically significant temporal differences were found within each group (*p* = 0.11 for uncoated; *p* = 0.87 for sphene-coated).

With regards to BIC%_Cancellous_, similar values were obtained in both the study arms, at 14- and 28-day healing periods (*p* = 0.95 and *p* = 0.99, respectively). As for BIC%, a non-significant trend toward an increase in BIC%_Cancellous_ over time within each group was identified (*p* = 0.21 for uncoated; *p* = 0.20 for sphene-coated).

In general, analyzing the healing pattern of the peri-implant bone in the two different bone compartments, cortical and cancellous, it is to be noted that apart from the sphene-coated samples at 28 days after the implant insertion, better bone–implant contact values were found in the cortical compartment.

**Figure 8:**
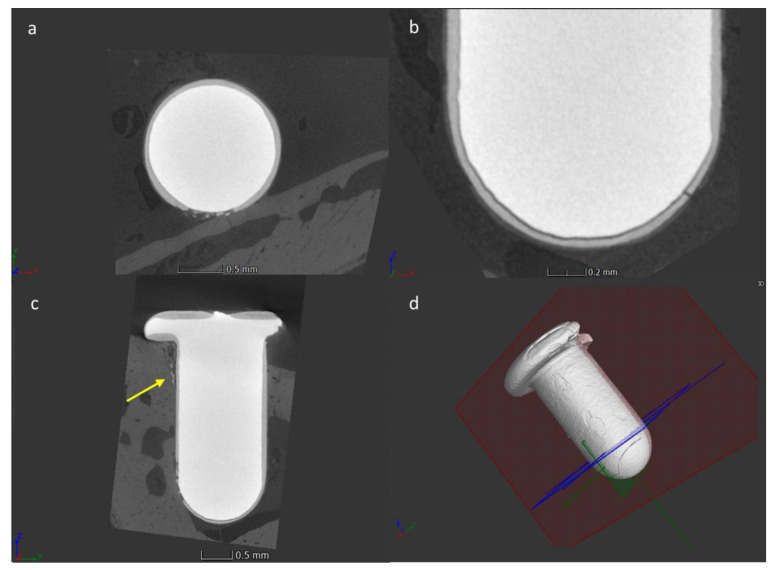
Micro-CT images of the sphene-coated implant presenting a partial delamination and disintegration of the bioceramic coating: (**a**) a transverse cross-sectional view; (**b**) a detail of the apical part of the implant; (**c**) a cross-sectional view parallel to the long axis of the implant, debris are indicated by the yellow arrow; (**d**) a 3D voxel-based reconstruction.

The coating delamination was observed in three samples, of which two at 28 days postoperatively and one after 14 days from surgery. In all these samples the delamination occurred in the transition zone between the cylindrical portion of the implant and the apical hemispherical one. Among these cases, in one case, along with the delamination of the coating, its disintegration determined the formation of particulate debris, which were detected in the cortical bone, close to an intact portion of the coating (Figure 8). This finding suggests that the cracking of the coating occurred during implant insertion.

**Figure 9:**
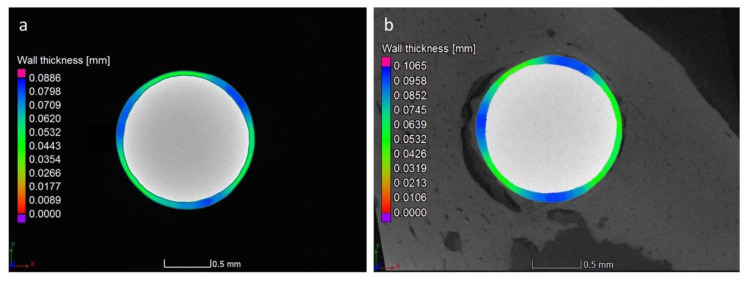
An example of the micro-CT transverse cross-sectional views of the sphene-coated implants with a coating thickness color map: (**a**) before the insertion; (**b**) positioned into the bone after 28 days of healing.

Finally, the mean values of the coating thickness at 14 and 28 days of healing were measured, and were found to be 63.4 ± 2.7 µm and 67 ± 2.7 µm, respectively. Representative micro-CT images with a color map indicating the thickness of the coating are provided in Figure 9. The lowest deviation after healing, compared to the implant before insertion, was due to the larger sample size.

### 3.5. Histological and Histomorphometrical Analyses

**Figure 10:**
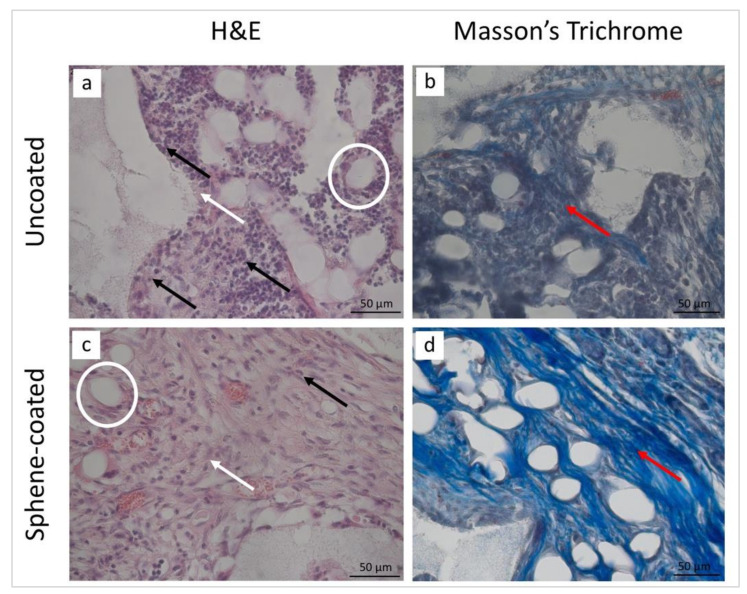
Staining with hematoxylin and eosin (H&E) (**a**,**c**) and Masson’s trichrome (**b**,**d**) of the peri-implant tissues around the uncoated (**a**,**b**) and the sphene-coated implants after 14 days of healing. In both experimental conditions, the inflammatory cells (black arrows) and fibroblasts (white arrows) were detected. A new extracellular matrix (ECM) rich in collagen fibers (red arrows) was observed, as well as the presence of several vessels (white circles).

**Figure 11:**
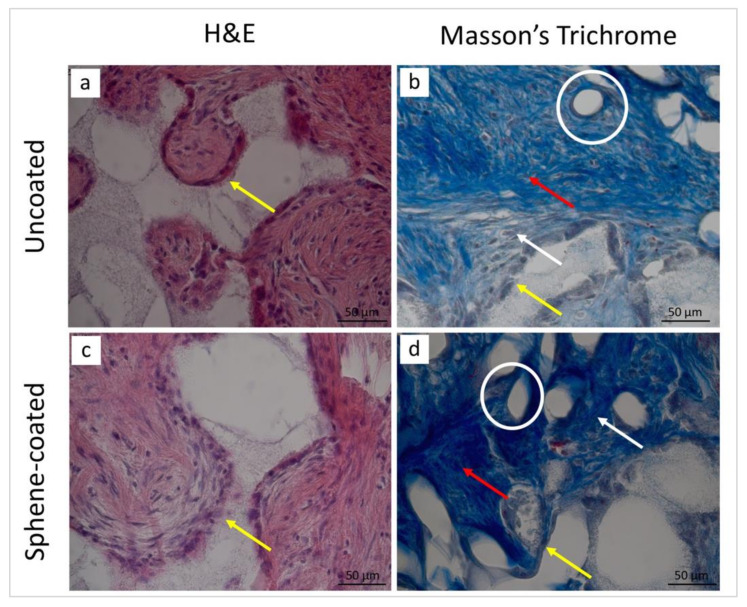
Staining with H&E (**a**,**c**) and Masson’s trichrome (**b**,**d**) of the peri-implant tissues around the uncoated (**a**,**b**) and the sphene-coated implants after 28 days of healing. In both experimental conditions, fibroblasts (white arrows), collagen fibers (red arrows), as well as vessels (white circles) could be observed. Moreover, osteogenic cells (yellow arrows) were detected.

The histological analysis was performed in order to observe the microstructure of the peri-implant tissues. The examination of the histological sections confirmed a similar healing pattern for the uncoated and sphene-coated implants. The representative images at 14 and 28 days after surgery are presented in Figure 10 and Figure 11, respectively.

After the 14-day healing period, in both groups the presence of inflammatory cells and fibroblasts, as well as new vessels, could be observed. In this early phase of healing, new bone formation could not be detected. Controversially, 28 days after surgery, in both the test and control conditions, the peri-implant tissues were characterized by the presence of vessels embedded in the extracellular matrix (ECM) and of osteogenic cells producing new bone matrix. At this stage, no sign of inflammatory process could be observed.

The histomorphometric analysis of the peri-implant tissues was performed and is reported in Table 6. Similar findings were obtained in both the test and control samples. Polymorphonuclear cells and phagocytic cells were abundant after 14 days and absent after 28 days from implantation. An increase in the number of fibroblasts and collagen fibers over time could be observed, while endothelial cells were moderately present at both time points. Finally, new bone formation was detected only 28 days postoperatively.

### 3.6. Synovial Fluid (SF) Analysis

Overall, SF was found in knee joints on the control side (6/10, 60%), but not on the test side (0/10, 0%), where the sphene-coated implants had been positioned (*p* = 0.04). The presence of SF on the control side was observed in the samples at both 14 days (4/5 [80%] in uncoated vs. 0/5 [0%] in sphene-coated, *p* = 0.13) and at 28 days (2/5 [40%] in uncoated vs. 0/5 [0%] in sphene-coated, *p* = 0.48).

When present, the volume of SF aspirate varied between 5 and 200 µl. It has to be noted that in 5 cases out of the 6 in which SF could be collected the total white blood cell count was equal to 100 WBC/mm^3^, and that was within the non-inflammatory range, whereas only one control sample, retrieved after 28 days of healing, presented with a clinically relevant amount of white blood cells (7000 WBC/mm^3^), albeit in absence of symptoms.

## 4. Discussion

The aim of the present in vivo study was to evaluate the performances of sphene-coated implants as compared to uncoated rough Ti implants in a rat femur model. The micro-CT analysis revealed slight differences, in terms of BIC measurements, between the two arms of the study at both time points. The differences in BIC values were lower than expected, therefore the study resulted as underpowered for demonstrating such differences. This might be due to the high-performance ZirTi implant surface utilized as a control. Consistently, histological and histomorphometrical findings were similar for both the uncoated and the sphene-coated implants, in which new bone formation was observed after 28 days from implant insertion. In addition, the present study confirmed that the sphene-based coating has no toxic or hemolytic activity, therefore it is safe to use in implant dentistry.

Previous studies have evaluated implant osseointegration using micro-CT imaging with encouraging results [31,32,33,34,35].

One of the major problems associated with the quantification of bone surrounding a Ti implant using micro-CT imaging, consists in the extent of the metal-induced artefacts, which might affect measurements in close proximity to the implants [60,61]. However, in a more recent study [33], a significant correlation was observed between the BIC assessed via backscatter scanning electron microscopy (bSEM) and micro-CT.

One of the major advantages of micro-CT is the possibility to use the whole 3D information of the bone–implant interface [33,62,63,64,65]. In a recent study in minipigs [65], in which a ROI based on a circular zone from 20 to 50 µm orthogonal to the implant surface was considered for BIC calculation, a statistically significant correlation was observed between micro-CT and histomorphometric analysis with regard to BIC.

In Choi et al. [66], an algorithm was used to calculate the 3D BIC area. The latter was obtained by dividing the attached bone volume by an infinitesimally increased radius (30 µm).

A similar approach was used in the present study. The simple geometry of the implants facilitated the calculation, which might become more complex in case of screw-type implants [66].

In the present study, the Ti implant with ZirTi surface was used as a reference as it is commonly used in clinical practice. Furthermore, the blasted and etched Ti surfaces generally demonstrated better results with respect to the machined ones when tested in animal models [67,68,69]. In Abron et al. [68], after three weeks of healing in rat tibiae, the BIC assessed via bSEM was significantly higher in the Ti implants with ideal pit morphology (BIC% 54 ± 7) than in the machined Ti implants (BIC% 34 ± 6) (*p* < 0.003).

Consistently with Abron et al. [68], using a similar animal model, in the present study the BIC assessed by a micro-CT of the uncoated Ti implants with sand-blasted and acid etched surface was of 48.1 ± 19.6% and 57.7 ± 15.4%, after two or four weeks of healing, respectively.

Although slightly higher BIC values were reported for sphene-coated samples (see Table 4), no statistically significant difference was found between the sphene-coated and uncoated samples at both time points (*p* > 0.05).

Micro-CT findings were confirmed by the histological and histomorphometric analyses, which demonstrated no marked differences in the healing patterns between the two implant surfaces. The bone healing after the implant insertion involved several phases, such as the activation of the immune system, neo-angiogenesis and new bone formation [70,71]. After 14 days from the implant insertion, the peri-implant tissues were characterized by the presence of inflammatory cells, fibroblasts and phagocytic cells, as well as by the apposition of new ECM and neo-angiogenesis. Twenty-eight days postoperatively, in both experimental conditions, no sign of inflammatory response could be observed, whereas newly formed bone was detected.

The biocompatibility of sphene coating was also demonstrated in another study, investigating the in vivo behavior of sol-gel derived sphene coatings [59]. Sphene-coated Ti-6Al-4V implants, produced by sol-gel and applied by dip-coating, were tested in vivo in the sheep femur model [59]. Six weeks after surgery, the histomorphometric analysis revealed that when positioned in cortico-cancellous bone, the sphene-coated implants showed a mean BIC of approximately 75%, comparable with the HA-coated ones, but significantly higher than that of the uncoated implants, with a BIC below 20%. These findings are in apparent contrast with what was reported in the present study, where no marked differences were observed between the control and test groups. This discrepancy might be due to the coating composition, that is made of only 31% sphene, the remaining composition being CaTiO_3_ and TiO_2_ [21]. This limitation in the amount of sphene is due to the fact that the synthesis of sphene in this work was made in situ on the implant and the heat treatment was limited to 950 °C, instead of 1350 °C, in order to avoid grain growth and the embrittlement of the metallic implant. Moreover, the animal models, as well as the implant designs and observation times were different in the two studies, therefore a direct comparison is not applicable.

In accordance with a recent study in rabbit tibiae [72], apart from in the sphene-coated implants after 28 days of healing, the osseointegration was higher, on average, in the cortical compartment compared to the cancellous one. However, as for BIC%, no statistically significant difference was found between the uncoated and coated implants with regards to both BIC%_Cortical_ and BIC%_Cancellous_.

Although the release of Ca and Si ions was found to stimulate osteoblastic proliferation and differentiation [73], one of the shortcomings of silicate-based ceramic coatings (i.e., wollastonite coatings) is their high degradation rate, which might affect their integrity [74,75].

When compared to other silicate bioceramics, the degradation kinetic of sphene ceramics tends to be very slow [13]. In previous investigations, sphene-coated samples produced using the same technique proposed in the present work resulted to be chemically stable in Tris-HCl [21]. Here, the mean coating thickness before and after implantation was assessed, confirming the absence of the rapid degradability of the proposed coating.

As for HA coatings [76], the delamination of the sphene-based coating from the substrate was found to be an issue of major concern. In a previous study, scratch tests were carried out on coated samples using two different solutions and deposited with an automated airbrush either for 1 s or 2.5 s [15]. The best results were obtained by using the same suspension composition and deposition time (1 s) used in the present study, with a critical normal load Lc 8.6 ± 0.2 N. Therefore, it was chosen to maintain the same composition and process parameters for the following in vitro [21] and then in vivo studies.

Contrary to previous works [15,21], in this study the coating was not applied onto flat plates, but onto cylindrical implants. As observed on micro-CT images, in all the three cases the delamination started in the transition zone between the cylindrical portion of the implant and the apical hemispherical one, involving the latter.

The weak adhesion of the coating in this area might be due to the deposition method, as in this portion of the implant the coating was not sprayed orthogonally to the surface. Additionally, the movement of the implant during the healing phases is regarded as one of the factors affecting implant osseointegration [77]. In this experimental study, the press fit of non-threaded implants into underprepared sites allowed for primary implant stability. It is likely that delamination occurred during the implant insertion, as suggested by the presence of debris in one sample in the cortical part of the bone. As revealed by a finite element analysis [78], when undersized drilling protocols are chosen, high strain values are measured in the cortical part of the bone and the press-fit phenomenon is largely dependent on the cortical bone. However, undersized drilling protocols can also be chosen in the clinical practice [79]. Even though lower stresses can be generated during implant insertion, it is necessary to improve the adhesion strength in any case.

Regarding the presence of SF, no aspirate could be collected from the joints on the side treated with the sphene-coated implants. When the uncoated implants were positioned, SF was detected in 6 cases. However, only in one case was the SF inflammatory, even though no other signs or symptoms were recorded. Taking everything into account, on one hand, these findings confirmed the biocompatibility of the proposed sphene-based coating whilst on the other hand, there were not sufficient data to determine the cause of the joint inflammation with certainty.

In conclusion, sphene ceramic is confirmed to be a biocompatible material for dental and orthopedic applications. However, further studies are needed to address the issue of coating delamination prior to clinical testing.

## Figures and Tables

**Table 1:** Suspension composition.

Component Compositions *	Amount (g)
SiO_2_ precursor “MK polymer”	3.65
CaCO_3_	5.12
TiO_2_	4.07
Isopropyl alcohol	14

*: The reported weight of components, in grams, is for obtaining the total ceramic yield of 10 g of sphene.

**Table 2:** Computed tomography (CT)-scanning parameters for the implants before the surgery.

Parameter	Value
X-ray source voltage	110 kV
X-ray source current	62 μA
Exposure time	1415 ms
Nr. of projections	3142
Voxel size	3 μm
Temperature	20 °C

**Table 3:** CT-scanning parameters used for investigating the bone osseointegration.

Parameter	Value
X-ray source voltage	170 kV
X-ray source current	40 μA
Exposure time	1415 ms
Nr. of projections	2000
Voxel size	6.5 μm
Temperature	20 °C

**Table 4:** Hemolysis assay

Sample	OD	HI	Results
Positive Control	0.8700 ± 0.012	100%	Hemolitic
Negative control	0.0134 ± 0.007	0%	Non-hemolitic
Sphene samples	0.0123 ± 0.002	0%	Non-hemolitic

OD, absorbance value at 540 nm: mean of three independent experiments ± SD; HI, hemolysis index.

**Table 5:** BIC%, BIC%_Cortical_ and BIC%_Cancellous_ values in the uncoated and the sphene-coated arms.

Sacrifice (days)	Arm (n implants)	BIC%	BIC%_Cortical_	BIC%_Cancellous_
14	Uncoated (5)	48.1 ± 19.6	44.6 ± 18.6	42.2 ± 18.4
	Sphene-coated (5)	50.5 ± 17.4	55.5 ± 16.1	41.5 ± 18.2
28	Uncoated (5)	57.7 ± 15.4	62.2 ± 9.4	56.2 ± 14.1
	Sphene-coated (5)	60.4 ± 17.7	53.9 ± 17.7	56.3 ± 14.8

Data are expressed as the mean ± standard deviation.

**Table 6:** Results of the histomorphometric measurements after 14 and 28 days from implantation for the uncoated and the sphene-coated implants.

	Uncoated		Sphene-coated	
	14 days	28 days	14 days	28 days
Polymorphonuclear cells	3	0	3	0
Phagocytic cells	3	0	3	0
Fibroblasts	1	2	1	2
Endothelial cells	2	2	2	2
Collagen fibers	2	3	2	3
New bone	0	2	0	2

0 = absence; 1 = scarcely present; 2 = moderately present; 3 = abundantly present.

## Supplementary Materials

The following are available online at https://www.mdpi.com/2077-0383/9/5/1290/s1, Figure S1: Representative micro-CT images of an uncoated Ti implant inserted in the rat femur with indications for the ROIs for calculation of BIC%_Cortical_ and BIC%_Cancellous_, Figure S2: Representative micro-CT images of a sphene-coated Ti implant inserted in the rat femur with indications for the ROIs for calculation of BIC%_Cortical_ and BIC%_Cancellous_.

## References

[1] Albrektsson T., Brånemark P.I., Hansson H.A., Lindström J. (1981). Osseointegrated titanium implants. Requirements for ensuring a long-lasting, direct bone-to-implant anchorage in man. Acta Orthop. Scand..

[2] Zreiqat H., Valenzuela S.M., Nissan B.B., Roest R., Knabe C., Radlanski R.J., Renz H., Evans P.J. (2005). The effect of surface chemistry modification of titanium alloy on signalling pathways in human osteoblasts. Biomaterials.

[3] Cordeiro J.M., Barão V.A. (2017). Is there scientific evidence favoring the substitution of commercially pure titanium with titanium alloys for the manufacture of dental implants?. Mater. Sci. Eng. C Mater. Biol. Appl..

[4] Pellegrini G., Francetti L., Barbaro B., Del Fabbro M. (2018). Novel surfaces and osseointegration in implant dentistry. J. Investig. Clin. Dent..

[5] Smeets R., Stadlinger B., Schwarz F., Beck-Broichsitter B., Jung O., Precht C., Kloss F., Gröbe A., Heiland M., Ebker T. (2016). Impact of dental implant surface modifications on osseointegration. Biomed. Res. Int..

[6] Brunello G., Elsayed H., Biasetto L. (2019). Bioactive glass and silicate-based ceramic coatings on metallic implants: Open challenge or outdated topic?. Materials.

[7] Xuereb M., Camilleri J., Attard N.J. (2015). Systematic review of current dental implant coating materials and novel coating techniques. Int. J. Prosthodont..

[8] Aebli N., Krebs J., Stich H., Schawalder P., Walton M., Schwenke D., Gruner H., Gasser B., Theis J.C. (2003). In vivo comparison of the osseointegration of vacuum plasma sprayed titanium- and hydroxyapatite-coated implants. J. Biomed. Mater. Res. A.

[9] Roy M., Bandyopadhyay A., Bose S. (2011). Induction plasma sprayed nano hydroxyapatite coatings on titanium for orthopaedic and dental implants. Surf. Coat. Technol..

[10] Wang H., Eliaz N., Xiang Z., Hsu H.P., Spector M., Hobbs L.W. (2006). Early bone apposition in vivo on plasma-sprayed and electrochemically deposited hydroxyapatite coatings on titanium alloy. Biomaterials.

[11] Artzi Z., Carmeli G., Kozlovsky A. (2006). A distinguishable observation between survival and success rate outcome of hydroxyapatite-coated implants in 5-10 years in function. Clin. Oral Implant. Res..

[12] Asri R.I., Harun W.S., Hassan M.A., Ghani S.A., Buyong Z. (2016). A review of hydroxyapatite-based coating techniques: Sol-gel and electrochemical depositions on biocompatible metals. J. Mech. Behav. Biomed. Mater..

[13] Wu C., Chang J. (2013). A review of bioactive silicate ceramics. Biomed. Mater..

[14] Garcia E., Miranzo P., Sainz M.A. (2018). Thermally sprayed wollastonite and wollastonite-diopside compositions as new modulated bioactive coatings for metal implants. Ceram. Int..

[15] Biasetto L., Elsayed H. (2017). Sphene silicate ceramic coatings on cpTi substrates: Process upgrade. Surf. Coat. Technol..

[16] Yi D., Wu C., Ma X., Ji H., Zheng X., Chang J. (2012). Preparation and in vitro evaluation of plasma-sprayed bioactive akermanite coatings. Biomed. Mater..

[17] Liang Y., Xie Y., Ji H., Huang L., Zheng X. (2010). Excellent stability of plasma-sprayed bioactive Ca_3_ZrSi_2_O_9_ ceramic coating on Ti–6Al–4V. Appl. Surf. Sci..

[18] Yi D., Wu C., Ma B., Ji H., Zheng X., Chang J. (2014). Bioactive bredigite coating with improved bonding strength, rapid apatite mineralization and excellent cytocompatibility. J. Biomater. Appl..

[19] Götz W., Tobiasch E., Witzleben S., Schulze M. (2019). Effects of silicon compounds on biomineralization, osteogenesis, and hard tissue formation. Pharmaceutics.

[20] Elsayed H., Colombo P. (2016). Crack-free silicate bioceramics from preceramic polymers. Adv. Appl. Ceram..

[21] Elsayed H., Brunello G., Gardin C., Ferroni L., Badocco D., Pastore P., Sivolella S., Zavan B., Biasetto L. (2018). Bioactive sphene-based ceramic coatings on cpTi substrates for dental implants: An in vitro study. Materials.

[22] Wu C., Ramaswamy Y., Liu X., Wang G., Zreiqat H. (2009). Plasma-sprayed CaTiSiO_5_ ceramic coating on Ti-6Al-4V with excellent bonding strength, stability and cellular bioactivity. J. R. Soc. Interface.

[23] Wang G., Lu Z., Liu X., Zhou X., Ding C., Zreiqat H. (2011). Nanostructured glass-ceramic coatings for orthopaedic applications. J. R. Soc. Interface.

[24] Li K., Yu J., Xie Y., Huang L., Ye X., Zheng X. (2011). Chemical stability and antimicrobial activity of plasma sprayed bioactive Ca_2_ZnSi_2_O_7_ coating. J. Mater. Sci. Mater. Med..

[25] Zhang W., Wang G., Liu Y., Zhao X., Zou D., Zhu C., Jin Y., Huang Q., Sun J., Liu X. (2013). The synergistic effect of hierarchical micro/nano-topography and bioactive ions for enhanced osseointegration. Biomaterials.

[26] Wu C., Ramaswamy Y., Soeparto A., Zreiqat H. (2008). Incorporation of titanium into calcium silicate improved their chemical stability and biological properties. J. Biomed. Mater. Res. A..

[27] Wu C., Ramaswamy Y., Gale D., Yang W., Xiao K., Zhang L., Yin Y., Zreiqat H. (2008). Novel sphene coatings on Ti-6Al-4V for orthopedic implants using sol-gel method. Acta Biomater..

[28] Biasetto L., Bertolini R., Elsayed H., Ghiotti A., Bruschi S. (2019). Use of cryogenic machining to improve the adhesion of sphene bioceramic coatings on titanium substrates for dental and orthopaedic applications. Ceram. Int..

[29] Wancket L.M. (2015). Animal models for evaluation of bone implants and devices: Comparative bone structure and common model uses. Vet. Pathol..

[30] Coelho P.G., Granjeiro J.M., Romanos G.E., Suzuki M., Silva N.R., Cardaropoli G., Thompson V.P., Lemons J.E. (2009). Basic research methods and current trends of dental implant surfaces. J. Biomed. Mater. Res. B Appl. Biomater..

[31] Vandeweghe S., Coelho P.G., Vanhove C., Wennerberg A., Jimbo R. (2013). Utilizing micro-computed tomography to evaluate bone structure surrounding dental implants: A comparison with histomorphometry. J. Biomed. Mater. Res. B Appl. Biomater..

[32] Stadelmann V.A., Potapova I., Camenisch K., Nehrbass D., Richards R.G., Moriarty T.F. (2015). In vivo MicroCT monitoring of osteomyelitis in a rat model. Biomed. Res. Int..

[33] Meagher M.J., Parwani R.N., Virdi A.S., Sumner D.R. (2018). Optimizing a micro-computed tomography-based surrogate measurement of bone-implant contact. J. Orthop. Res..

[34] He T., Cao C., Xu Z., Li G., Cao H., Liu X., Zhang C., Dong Y. (2017). A comparison of micro-CT and histomorphometry for evaluation of osseointegration of PEO-coated titanium implants in a rat model. Sci. Rep..

[35] Yu Y.J., Zhu W.Q., Xu L.N., Ming P.P., Shao S.Y., Qiu J. (2019). Osseointegration of titanium dental implant under fluoride exposure in rabbits: Micro-CT and histomorphometry study. Clin. Oral Implant. Res..

[36] Geng H., Todd N.M., Devlin-Mullin A., Poologasundarampillai G., Kim T.B., Madi K., Cartmell S., Mitchell C.A., Jones J.R., Lee P.D. (2016). A correlative imaging based methodology for accurate quantitative assessment of bone formation in additive manufactured implants. J. Mater. Sci. Mater. Med..

[37] Chikazu D., Tomizuka K., Ogasawara T., Saijo H., Koizumi T., Mori Y., Yonehara Y., Susami T., Takato T. (2007). Cyclooxygenase-2 activity is essential for the osseointegration of dental implants. Int. J. Oral Maxillofac. Surg..

[38] Liu W., Zhang S., Zhao D., Zou H., Sun N., Liang X., Dard M., Lanske B., Yuan Q. (2014). Vitamin D supplementation enhances the fixation of titanium implants in chronic kidney disease mice. PLoS ONE.

[39] Zheng X., Mo A., Wang Y., Guo Y., Wu Y., Yuan Q. (2017). Effect of FK-506 (tacrolimus) therapy on bone healing of titanium implants: A histometric and biomechanical study in mice. Eur. J. Oral Sci..

[40] Pelegrine A.A., Moy P.K., Moshaverinia A., Escada A.L.D.A., Calvo-Guirado J.L., Claro A.P.R.A. (2019). Development of a novel nanotextured titanium implant. An experimental study in rats. J. Clin. Med..

[41] Zhou W., Tangl S., Reich K.M., Kirchweger F., Liu Z., Zechner W., Ulm C., Rausch-Fan X. (2019). The Influence of type 2 diabetes mellitus on the osseointegration of titanium implants with different surface modifications-A histomorphometric study in high-fat diet/low-dose streptozotocin-treated rats. Implant Dent..

[42] Courtney P., Doherty M. (2013). Joint aspiration and injection and synovial fluid analysis. Best Pr. Res. Clin. Rheumatol..

[43] Punzi L., Oliviero F. (2009). Arthrocentesis and synovial fluid analysis in clinical practice: Value of sonography in difficult cases. Ann. N. Y. Acad. Sci..

[44] Strzelec-Nowak D., Kozioł-Montewka M., Niedźwiadek J., Bogut A., Blacha J., Mazurkiewicz T. (2015). Investigation of the actual causes of hip joint implant loosening classified as aseptic--analysis of microbiological culture results and levels of inflammatory markers. Pol. J. Microbiol..

[45] Delecrin J., Oka M., Takahashi S., Yamamuro T., Nakamura T. (1994). Changes in joint fluid after total arthroplasty. A quantitative study on the rabbit knee joint. Clin. Orthop. Relat. Res..

[46] Lin T., Tamaki Y., Pajarinen J., Waters H.A., Woo D.K., Yao Z., Goodman S.B. (2014). Chronic inflammation in biomaterial induced periprosthetic osteolysis: NF-κB as a therapeutic target. Acta Biomater..

[47] Takei I., Takagi M., Ida H., Ogino T., Santavirta S., Konttinen Y.T. (2000). High macrophage-colony stimulating factor levels in synovial fluid of loose artificial hip joints. J. Rheumatol..

[48] Fritz J., Lurie B., Potter H.G. (2015). MR imaging of knee arthroplasty implants. Radiographics.

[49] Ferraris S., Bobbio A., Miola M., Spriano S. (2015). Micro- and nano-textured, hydrophilic and bioactive titanium dental implants. Surf. Coat. Technol..

[50] ISO 25178-2 (2012). GPS—Surface Texture: Areal—Part 2: Terms, Definitions and Surface Texture Parameters.

[51] Kilkenny C., Browne W., Cuthill I.C., Emerson M., Altman D.G. (2011). National Centre for the Replacement, Refinement and Reduction of Amimals in Research. Animal research: Reporting in vivo experiments—The ARRIVE guidelines. J. Cereb. Blood Flow Metab..

[52] Scanu A., Oliviero F., Ramonda R., Frallonardo P., Dayer J.M., Punzi L. (2012). Cytokine levels in human synovial fluid during the different stages of acute gout: Role of transforming growth factor β1 in the resolution phase. Ann. Rheum. Dis..

[53] Al Farraj Aldosari A., Anil S., Alasqah M., Al Wazzan K.A., Al Jetaily S.A., Jansen J.A. (2014). The influence of implant geometry and surface composition on bone response. Clin. Oral Implant. Res..

[54] Buser D., Schenk R.K., Steinemann S., Fiorellini J.P., Fox C.H., Stich H. (1991). Influence of surface characteristics on bone integration of titanium implants. A histomorphometric study in miniature pigs. J. Biomed. Mater. Res..

[55] Eom T.G., Jeon G.R., Jeong C.M., Kim Y.K., Kim S.G., Cho I.H., Cho Y.S., Oh J.S. (2012). Experimental study of bone response to hydroxyapatite coating implants: Bone-implant contact and removal torque test. Oral Surg. Oral Med. Oral Pathol. Oral Radiol..

[56] Faeda R.S., Spin-Neto R., Marcantonio E., Guastaldi A.C., Marcantonio E. (2012). Laser ablation in titanium implants followed by biomimetic hydroxyapatite coating: Histomorphometric study in rabbits. Microsc. Res. Tech..

[57] Todisco M., Trisi P. (2006). Histomorphometric evaluation of six dental implant surfaces after early loading in augmented human sinuses. J. Oral Implant..

[58] Weinlaender M., Beumer J., Kenney E.B., Lekovic V., Holmes R., Moy P.K., Plenk H. (2006). Histomorphometric and fluorescence microscopic evaluation of interfacial bone healing around 3 different dental implants before and after radiation therapy. Int. J. Oral Maxillofac. Implant..

[59] Ramaswamy Y., Wu C., Dunstan C.R., Hewson B., Eindorf T., Anderson G.I., Zreiqat H. (2009). Sphene ceramics for orthopedic coating applications: An in vitro and in vivo study. Acta Biomater..

[60] Butz F., Ogawa T., Chang T.L., Nishimura I. (2006). Three-dimensional bone-implant integration profiling using micro-computed tomography. Int. J. Oral Maxillofac. Implant..

[61] Liu S., Broucek J., Virdi A.S., Sumner D.R. (2012). Limitations of using micro-computed tomography to predict bone-implant contact and mechanical fixation. J. Microsc..

[62] Bernhardt R., Kuhlisch E., Schulz M.C., Eckelt U., Stadlinger B. (2012). Comparison of bone-implant contact and bone-implant volume between 2D-histological sections and 3D-SRµCT slices. Eur. Cell. Mater..

[63] Jimbo R., Coelho P.G., Vandeweghe S., Schwartz-Filho H.O., Hayashi M., Ono D., Andersson M., Wennerberg A. (2011). Histological and three-dimensional evaluation of osseointegration to nanostructured calcium phosphate-coated implants. Acta Biomater..

[64] Palmquist A., Shah F.A., Emanuelsson L., Omar O., Suska F. (2017). A technique for evaluating bone ingrowth into 3D printed, porous Ti6Al4V implants accurately using X-ray micro-computed tomography and histomorphometry. Micron.

[65] Bissinger O., Probst F.A., Wolff K.D., Jeschke A., Weitz J., Deppe H., Kolk A. (2017). Comparative 3D micro-CT and 2D histomorphometry analysis of dental implant osseointegration in the maxilla of minipigs. J. Clin. Periodontol..

[66] Choi J.Y., Park J.I., Chae J.S., Yeo I.L. (2019). Comparison of micro-computed tomography and histomorphometry in the measurement of bone-implant contact ratios. Oral Surg. Oral Med. Oral Pathol. Oral Radiol..

[67] Sivolella S., Bressan E., Salata L.A., Urrutia Z.A., Lang N.P., Botticelli D. (2012). Osteogenesis at implants without primary bone contact—An experimental study in dogs. Clin. Oral Implant. Res..

[68] Abron A., Hopfensperger M., Thompson J., Cooper L.F. (2001). Evaluation of a predictive model for implant surface topography effects on early osseointegration in the rat tibia model. J. Prosthet. Dent..

[69] Marinho V.C., Celletti R., Bracchetti G., Petrone G., Minkin C., Piattelli A. (2003). Sandblasted and acid-etched dental implants: A histologic study in rats. Int. J. Oral Maxillofac. Implant..

[70] Terheyden H., Lang N.P., Bierbaum S., Stadlinger B. (2012). Osseointegration—Communication of cells. Clin. Oral Implant. Res..

[71] Bressan E., Botticelli D., Sivolella S., Bengazi F., Guazzo R., Sbricoli L., Ricci S., Ferroni L., Gardin C., Velez J.U. (2015). Adipose-derived stem cells as a tool for dental implant osseointegration: An experimental study in the dog. Int. J. Mol. Cell. Med..

[72] Soto-Peñaloza D., Caneva M., Viña-Almunia J., Martín-de-Llano J.J., Peñarrocha-Oltra D., Peñarrocha-Diago M. (2018). Bone-healing pattern on the surface of titanium implants at cortical and marrow compartments in two topographic sites: An experimental study in rabbits. Materials.

[73] Fei L., Wang C., Xue Y., Lin K., Chang J., Sun J. (2012). Osteogenic differentiation of osteoblasts induced by calcium silicate and calcium silicate/β-tricalcium phosphate composite bioceramics. J. Biomed. Mater. Res. B Appl. Biomater..

[74] Wu C., Ramaswamy Y., Kwik D., Zreiqat H. (2007). The effect of strontium incorporation into CaSiO3 ceramics on their physical and biological properties. Biomaterials.

[75] Ni S., Chang J. (2009). In vitro degradation, bioactivity, and cytocompatibility of calcium silicate, dimagnesium silicate, and tricalcium phosphate bioceramics. J. Biomater. Appl..

[76] Ogiso M., Yamashita Y., Matsumoto T. (1998). The process of physical weakening and dissolution of the HA-coated implant in bone and soft tissue. J. Dent. Res..

[77] Albrektsson T., Albrektsson B. (1987). Osseointegration of bone implants. A review of an alternative mode of fixation. Acta Orthop. Scand..

[78] Frisardi G., Barone S., Razionale A.V., Paoli A., Frisardi F., Tullio A., Lumbau A., Chessa G. (2012). Biomechanics of the press-fit phenomenon in dental implantology: An image-based finite element analysis. Head Face Med..

[79] Ostman P.O., Hellman M., Sennerby L. (2005). Direct implant loading in the edentulous maxilla using a bone density-adapted surgical protocol and primary implant stability criteria for inclusion. Clin. Implant Dent. Relat. Res..

