# Supplementary material for: An In Vivo Study in Rat Femurs of Bioactive Silicate Coatings on Titanium Dental Implants
Source: J Clin Med. 2020 Apr 29;9(5):1290. doi: 10.3390/jcm9051290 (PMC7288333; doi:10.3390/jcm9051290)
Supplement: Supplementary file 1 [file jcm-09-01290-s001.zip › Suppl_in vivo sphene_J Clin Med_23_02_2020.docx]

**SUPPLEMENTARY MATERIAL**


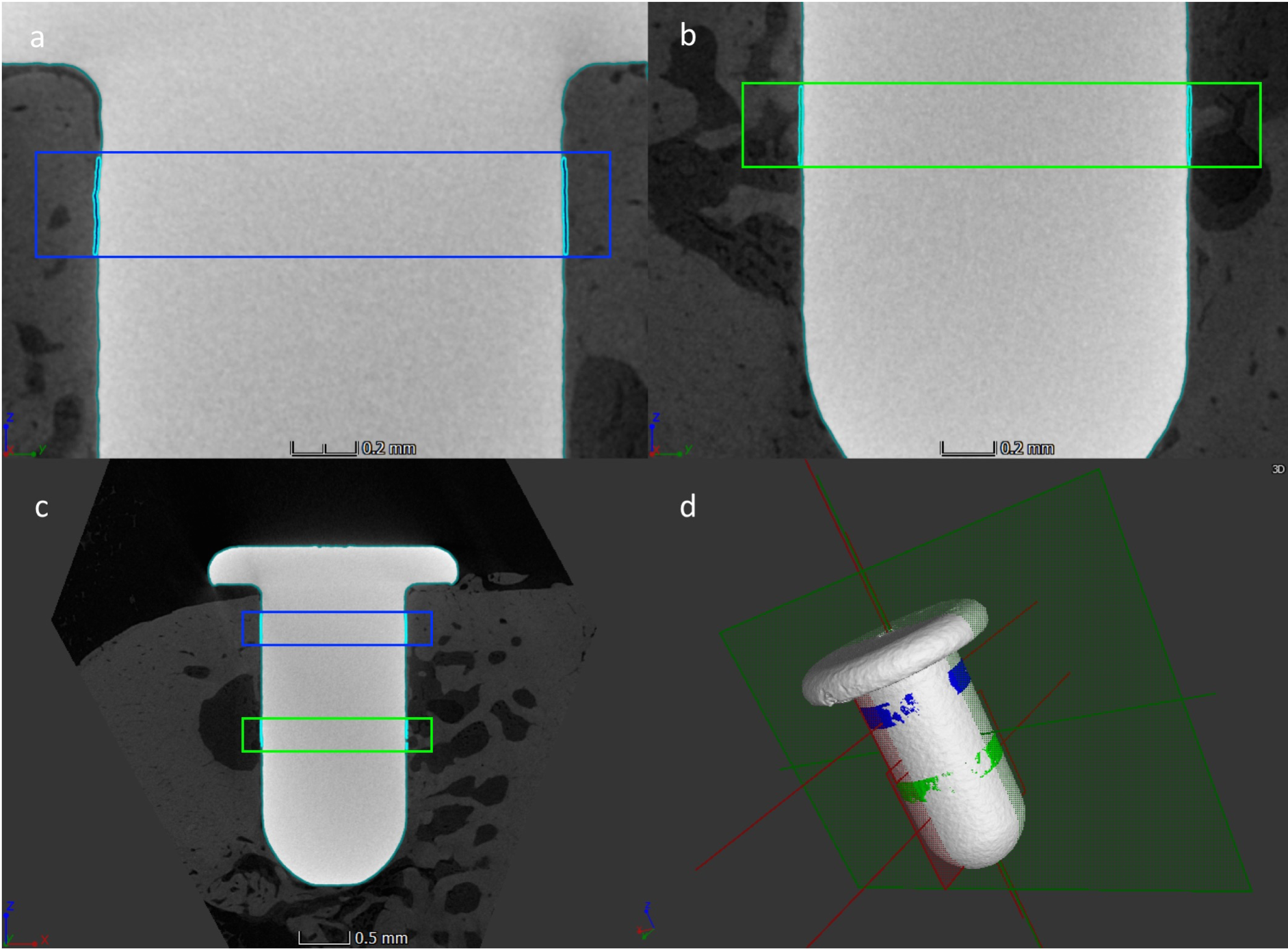


**Figure S1.** Representative micro-CT images of an uncoated Ti implant inserted in the rat femur after 28 days of healing with indications in blue and green for the ROIs for calculation of BIC%_Cortical_ and BIC%_Cancellous_, respectively. In light blue the intersection volume between the ROI_I+1_ and ROI_B_ in the portions considered for calculation. (a) Detail of the cortical part; (b) detail of cancellous part; (c) cross-sectional view parallel to the long axis of the implant; (d) 3D voxel-based reconstruction of the uncoated implant.


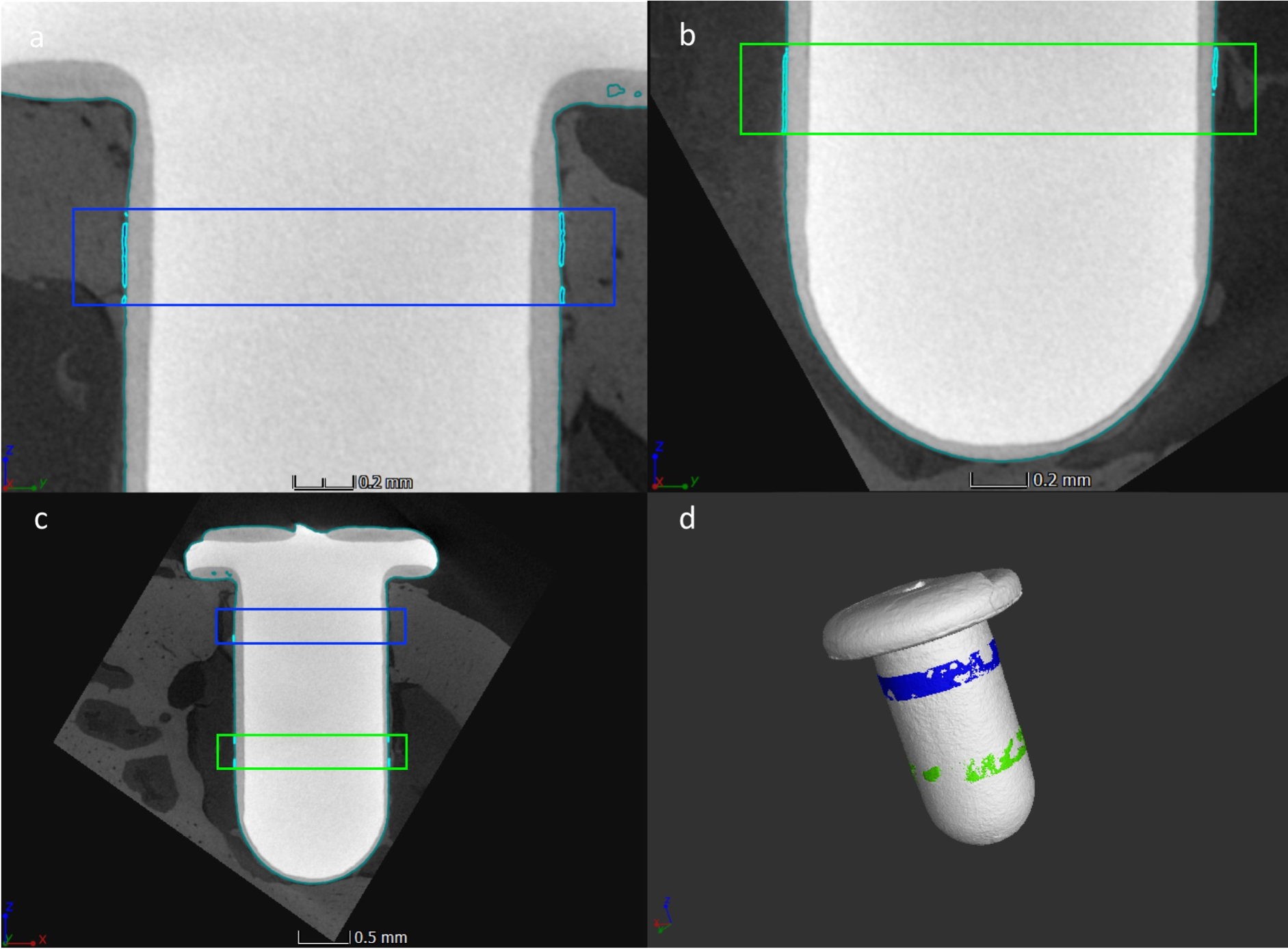


**Figure S2.** Representative micro-CT images of sphene-coated Ti implant inserted in the rat femur after 28 days of healing with indications in blue and green for the ROIs for calculation of BIC%_Cortical_ and BIC%_Cancellous_, respectively. In light blue the intersection volume between the ROI_I+C+1_ and ROI_B_ sin the portions considered for calculation. (a) Detail of the cortical part; (b) detail of cancellous part; (c) cross-sectional view parallel to the long axis of the implant; (d) 3D voxel-based reconstruction of the sphene-coated implant.
